# Revealing an enabling environment: How clinical community and clinical stakeholders understand peer navigation to improve quality of life for people living with HIV in Australia

**DOI:** 10.3389/fpubh.2023.1101722

**Published:** 2023-04-20

**Authors:** Timothy Krulic, Graham Brown, Sara Graham, Jennifer Hoy, Adam Bourne

**Affiliations:** ^1^Australian Research Centre in Sex, Health and Society, La Trobe University, Melbourne, VIC, Australia; ^2^Living Positive Victoria, Melbourne, VIC, Australia; ^3^The Centre for Social Impact, UNSW, Sydney, NSW, Australia; ^4^Alfred Hospital Infectious Diseases Clinic, Melbourne, VIC, Australia

**Keywords:** HIV, peer navigation, quality of life, stigma and discrimination, qualitative, implementation

## Abstract

People living with HIV have unique resources to offer each other and health systems. This study investigated how peer navigation might contribute to a socially supportive, health enabling environment in Victoria, Australia. We used semi-structured interviews with 30 program staff, management, peer workers and clinician stakeholders. Our analyses considered the interplay between the program, users, HIV-related stigma and discrimination and the health service environment. Peer relationships offered reassurance, acceptance and belonging, which people living with HIV can use to create personal change. Peer engagement coproduced insights for life with HIV and may help to overcome stigma and structural barriers to access services and community support. As a partnership between peer and clinical services, participants described how the program fostered appreciation of peer practices and insights, which were used to improve the quality and continuity of care offered in the state. These findings allude to the value of the community engagement and policy alignment peer responses produce and can be used to guide implementation of similar programs elsewhere.

## 1. Introduction

A community and peer response to HIV has long existed in Australia. The communities most affected by HIV and AIDS, including people living with HIV, mobilised early in the epidemic to participate in leadership, policy development and the promotion of awareness and prevention practices (1, 2). Such partnerships are now recognised globally as critical to the success of any HIV response (3, 4).

Much of the focus of Australia’s peer response has shifted to support people who are HIV positive to live their lives to their full potential. Stigma, discrimination and intersecting health inequities remain persistent problems, despite biomedical advances in treatment and prevention, (5–8). Reaching beyond clinical outcomes to improve quality of life is now a major focus of the Australian response (9). Ongoing investment in the delivery, evaluation, and adaptation of peer programs has, however, been somewhat fragmented (1, 10).

Peer-based support and approaches take many forms. Underpinning the peer approach is the knowledge that people from similar communities, backgrounds, and experiences have unique resources to offer each other (11). At its core, peer support is a system of giving and receiving help to bring about desired personal or social change, grounded in principles of reciprocity, mutual respect, and equality (12). Supportive peer relationships can exist within naturally occurring networks, self-help and support groups or community controlled and operated services. A growing body of research positions peers providing health systems navigation and support through complex issues and service environments as a more formal occupation for people living with HIV working alongside healthcare practitioners (13, 14).

Peer navigation and support have been associated with a wide range of benefits. Peers supporting people experiencing severe mental health challenges have shown promising increases in quality of life, self-esteem and efficacy, social connectedness, empowerment and engagement in treatment, services and community (15). Research investigating peer interventions for people living with HIV, however, has tended to focus on individual behavioural effects related to specific continuum of care outcomes (16). A recent scoping review shows that there has been little research to understand how HIV peer navigation programs can influence culturally and contextually informed assessments of health, such as quality of life (14).

The purpose of this article is to explore how peer navigation programs might contribute to a socially supportive, health enabling environment for people living with HIV. Moore and Dietze used the concept of the enabling environment to reframe discussions of reducing drug-related harm often defined by strategies focused at the level of the individual (17). They advocate for placing greater emphasis on addressing the social and environmental determinants that facilitate or impede behaviour change. We similarly seek to recognise that an individual’s quality of life is influenced not only by their behaviour, but also by community, care, support and service environments. Within this frame our study aimed to better understand how peer navigated guidance may mediate or facilitate personal, social and service change that improves quality of life people living with HIV. We also seek to acknowledge that peer programs engage and influence communities as part of and in collaboration with them (18). Observing how a peer program learns, adapts, and aligns with the HIV service environment in response to its engagement with its social system can capture its wider impact and elicit practical knowledge to guide many aspects of program delivery, partnership, and policy development (19). Our analyses consider the nature of peer engagement and the interplay between the program, users, the people living with HIV community, HIV-related stigma and discrimination and the health service environment so that findings may be used to improve the impact and implementation of similar programs.

## 2. Materials and methods

### 2.1. Research setting and program description

This article reports findings from an implementation study of an HIV peer navigation program operating in Victoria, Australia. Living Positive Victoria, a community-controlled organisation representing people living with HIV, established the peer navigation program in 2018.

Unlike other peer programs operating in Victoria, peer navigator positions were funded to respond to requests for tailored, in-person support from clinical partners. Navigators had broad scope to support clients across any health issue or quality of life concern they had in relation to HIV. Individual sessions lasted an hour or so, but client access to the program was not limited.

Three people living with HIV were recruited as navigators. Living Positive Victoria selected navigators with skills and experiences demonstrated through engagement with diverse communities of people living with HIV in Victoria. This includes women and heterosexual men, gay and bisexual men and men who have sex with men, and people from migrant backgrounds. Roles were part-time and paid in-line with national skills-based awards for social and community work. Navigators received training and supervision from Living Positive Victoria.

The program established formal referral relationships with five major treatment centres in Melbourne. Partner clinics spanned two general practices, two hospital-based infectious disease clinics and the only large, publicly funded sexual health clinic available to all people in the state at no cost.

### 2.2. Participants and approach

Our approach drew on community participatory research principles. The delivery of the peer navigation program was driven by the participating community organisation and its partner clinics rather than the academic members of the research team. Representatives from these organisations assisted in the design of appropriate research methods, participant recruitment, the evaluation of findings and authorship of journal articles. Findings involving the participation of service users will be reported elsewhere.

This article draws on engagement with 30 staff and representatives from Living Positive Victoria and its partner clinics. This included three peer navigators, four members of leadership and management, and four peer workers delivering other initiatives at Living Positive Victoria. Among the clinician stakeholders were five infectious disease specialists, eight nurses and clinical support staff, and two nurse practitioners who worked across the two public hospitals and the sexual health clinic. Four general practitioners (GPs), two from each clinic, participated in the study. The infectious disease and general practitioners included members of clinic management and casuals who worked fractional hours across several partner clinics.

We selected study participants who had close working knowledge of the peer navigation program. As peer workers, program staff and clinical stakeholders, participants were aware of the care and support needs of a range of different communities of people living with HIV in Victoria as well as contextual factors relevant to the program’s success. Key program staff and partners were identified through meetings with program management and invited to participate directly by the research team. A wider invitation to all staff who had knowledge or involvement with the program was sent by management in email communications.

Participants provided written and oral informed consent. Ethical approval was obtained from the La Trobe University (HEC19033) and Alfred Health (HREC53336).

### 2.3. Data collection and analysis

In this article we draw on data collected in interviews and focus groups held June 2019 to February 2020. These provided detailed accounts of the peer navigation program’s operation and how it learned, adapted, and aligned with the HIV service environment in response to its engagement with its social system.

The first author held four focus group discussions (FGD) with larger staff groups of peer navigators, other peer workers, and nurses and clinical support staff working across two separate clinics. Interviews were held in person or over the phone with infectious disease specialists, nurse practitioners, GPs, and members of leadership and management at Living Positive Victoria.

Interviews and focus groups were semi-structured. The interviewer asked participants to describe their involvement with the program and how they believe it operated to effect changes in the HIV care and service environment and the health and wellbeing of service users. Participants were then asked if there were any recommendations they would make, or lessons learned from implementing the program.

The first author transcribed and thematically analysed data in a manner similar to that described by Braun and Clarke (20). Interviews were recorded, transcribed, read and reread after which codes and categories were developed. The study team further refined codes and categories into themes with attention paid to commonalities and connections across the sample, and implications within the broader research literature. Feedback from representatives of Living Positive Victoria and clinical stakeholders guided the evaluation of our findings.

## 3. Findings

Our findings indicate that the peer navigation relationship could produce insights and a sense of acceptance, belonging and reassurance that facilitated connection to services and community contexts that enable better quality of life. These themes are explored with consideration to the quality of peer engagement and the inhibiting effects of stigma on health and help-seeking behaviour for socially isolated people living with HIV. A fourth theme, the missing link, then considers how collaboration between clinical and peer providers contributed to the quality and continuity of care and support services.

### 3.1. Finding acceptance, belonging, and reassurance within peer navigation relationships

Participants from all groups spoke to the isolating effects of HIV stigma. Fearing or holding stigma was believed to prevent people living with HIV from seeking help and support from friends, family, partners, and peer services. As one GP explained, “we do meet people who six years, twelve years on have still not disclosed to more than one or two people or no-one even.” Culturally diverse new and temporary migrants, including gay and bisexual men and people from low prevalence groups, such as heterosexual men and women, were identified as the people living with HIV most likely to experience long-term isolation and its effects on self-esteem and wellbeing.

Support from peer navigators was positioned as a safe and effective way to help clients feel accepted and connected as a person living with HIV. Most participants said that simply meeting or befriending another person living with HIV could foster a sense of belonging. Feelings of being accepted, cared for, valued and understood were believed to be enhanced by peers who could demonstrate credible insight into personal struggles. Focus group discussions with navigators suggested that the dynamic of peer support encouraged such displays of struggle, empathy, and understanding. As one peer navigator explained, listening to the stories of clients is augmented by sharing.

With my clients there’s definitely a lot of back and forth and sharing of personal experiences and just relating as a human being. I don’t try to separate myself from my crew. My life is as messy as yours sometimes, my life is as great as yours sometimes. I do a lot of that, there's a lot of talking and sharing. And listening [peer navigator FGD].

Peer workers, program staff and management expected exchanges in which peers “relate … as human beings” to challenge negative perceptions of people living with HIV and provide a sense of normalcy. Peer workers also said that navigators offered a model of living proudly with HIV which clients could use to transform their own self-image. One peer navigator who grew up in a South Asian culture where homosexuality and HIV were heavily stigmatised explained that having this shared experience with some clients helped him to identify when they struggled with similar issues and challenge world views within the safety of the peer relationship.

Participants across all groups expected navigators who modelled a journey from diagnosis to living well with HIV to reduce concerns people experienced in relation to their own health, wellbeing, and lifegoals. Aspirations included having successful relationships and careers, children, or migrating to Australia. This sentiment was captured in a discussion between two nurses in a focus group from one clinic,

The proof is in the pudding really, like we can talk our heads off about how you're going to be fine, but if they actually meet someone who is…

Who is fine yeah?

And who is not only fine but is thriving.

Yeah.

That’s, I think, that’s much more powerful than any nurse or doctor telling them they're going to be fine.

Confirming treatment information from another trusted source was highly valued by clinicians, who expected that newly diagnosed people might not retain information provided at diagnosis.

### 3.2. Peers coproduce insights for life with HIV

Participants from all groups consistently conveyed the belief that peer navigators are the experts in life with HIV. Contemporary issues participants thought to influence the quality of life experienced by clients of the program and people living with HIV in Victoria more broadly included managing treatment and health concerns, migrating with HIV, disclosure and maintaining a fulfilling sex life and relationships with partners, friends, and family. Participants from all groups expected peer navigators to have practical and relevant advice based on their own experiences. Peer workers and program staff were clear that navigators create these insights collaboratively, within peer relationships, the people living with HIV community and the peer response to HIV.

In a focus group, two peer navigators talked about different approaches towards helping clients with problems.

You actually act as a sounding board for them, so they sort of bounce off ideas that they themselves have and how they want to approach their challenges, and it’s like they almost figure the answer out for themselves in that session. We pretty much listen to them. Although it helps if you know what to say in response.

I don’t know. I think it’s a lot of information sharing as well. That listening is important of course, but it’s having that space where they can come that most people don’t have anywhere else, and then of course like it’s a lot of talking and sharing of information, and you know where can you go? What can you do? What do we have?

As this exchange shows, navigators described a collaborative process to find tailored solutions based on the personal preferences, contexts and values of individual people living with HIV. As the second navigator clarifies, “listening is important” to realise this goal, but they were readily able provide information, resources and advice to guide which steps to take next.

Some peer insights came directly from navigators’ own life experiences. When advising clients on the process for securing visas and residency in Australia, one peer navigator said that they recommend a knowledgeable and trusted migration agent who supported their partner’s successful application for a visa. They were able to talk to clients about the most effective strategies to overcome discrimination and succeed in other pathways to residency because many other peers and clients had produced these insights together. Another peer navigator spoke of discussions with men from East, South, and Southeast Asia about preserving close connections with family while being attracted to other men, living with HIV and exploring gay or bi identity in Australia. His own experience taught him that it was possible to keep family ties intact while holding an empowered sense of himself as a gay man living with HIV from South Asian heritage. In his conversations with many men from similar backgrounds he emphasised personal choice and the validity of protecting oneself from experiencing rejection or discrimination by not revealing HIV status to friends and family.

Program staff and peer workers were clear that peer navigator expertise was enhanced by knowledge the peer response in Victoria had gathered through its long-term engagement with the community. One peer navigator elaborated on this claim made by another in a focus group discussion.

I think [peer navigation] is what Living Positive Victoria is about. Peer support is what it's always been doing, and for that 30 years it’s been around it’s always been about engaging the community, about supporting the community. It’s like what [they] said. It’s a reflection of that. It’s an extension of that.

Peer workers and program staff, including peer leadership and management, maintained that inviting program participants to collaborate in this way as peers, partners and community members builds confidence to meet challenges and other threats to health and wellbeing that may arise for the rest of their life with HIV.

### 3.3. Supporting connection to service and community environments

Participants spoke of how peer navigators helped clients to build connections that would enable sustained health and wellbeing. Often, these relationships were with healthcare practitioners and other social services.

Navigators had a wider remit than participants associated with the peer support previously available in Victoria. They provided support and referral to other services for issues such as anxiety, depression, alcohol, and other drug use, financial and housing insecurity, migration to Australia and complexities related to health and ageing with HIV. Peer navigators explained how these challenges intersected with HIV, and how clients benefited from the support and connections they could provide. For example, one peer navigator explained in a focus group discussion that she accompanied a woman to a medical appointment related to her breast health. “Of course, HIV is going to come up in that appointment,” she added. Given that the client had not told anybody about her HIV status, she could not bring a friend to support her with a potentially distressing or complex healthcare appointment. The peer navigator’s support, however, encouraged her to meet this challenge.

Other issues, such as drug use that was perceived as harmful, were described by peer navigators as pressing needs that once addressed would allow service users to focus on living well. As one peer navigator explained,

[sometimes] you’re just trying to get people to see harm reduction … they’re dealing with a lot of drug use and it’s hard to do an appointment [for] another thing. I know if I say here’s that number give them a call, it’s never going to happen. I’m happy to call them, make an appointment time, get them to that appointment, because sometimes people just need that push [peer navigator FGD].

Here, the navigator identifies harm reduction as a safe and non-judgmental approach towards managing harmful drug use, recognising that those who are unable to stop or comfortable with their use can still make positive change to protect themselves and others. Peers believed that they were skilled at identifying a level of support that would still enable clients to make their own choices about how to address their problems, “sometimes people just need that push.” Navigators said that these skills came from their own experiences as service users, insights shared by experienced peers working at Living Positive Victoria and training the organisation identified.

Ultimately, the relationships participants from all groups valued most were the connections peer navigators helped to forge between socially isolated clients and the community of people living with HIV in Victoria. Peer navigators spoke of how they provided introductions to community members or helped clients to access support groups and services where they would meet other people living with HIV. Participants from all groups thought that meeting a peer navigator first reduced the anxiety newly diagnosed or socially isolated people felt about identifying as HIV positive in social settings, even among peers. At other times, navigators accompanied clients to the start of workshops, facilitated introductions with peer workers or agreed to meet clients at social events.

Community environments were where peer workers and program staff and management hoped people living with HIV could build their own support networks. All participants groups agreed that increasing access to this level of social support would enable wellbeing and have flow on effects on engagement with care and quality of life. Peer workers and program staff argued that the egalitarian nature and interdependency of these relationships fostered empowerment and resilience. Peer workers and program staff hoped that the clients of the program could go on to be active participants in support groups and networks, take on volunteer roles in peer education, public speaking or other policy and community development roles in an HIV response where their perspectives, skills, and contributions were valued.

### 3.4. The “missing link”: Mediating clinical care

Clinician stakeholders described how the program fostered appreciation of peer practices and insights, which could be used to improve the quality and continuity of care offered in the state.

The program created opportunities for peer navigators and clinic staff to learn about each other. Peer workers said that previously, they were stretched to meet regularly with the nurses and doctors they relied on to promote their programs. Management organised meetings where peer navigators told staff groups about their backgrounds, training and stories of living with HIV. This information was distributed to the nurses, doctors and clinical support staff most involved with care coordination and referring clients to the program.

At sites where peer navigators provided clinic-based appointments or remained on site during clinic hours, staff had opportunities to talk and observe each other at work. A medical practitioner at one clinic explained in his interview that this level of familiarity, “getting to know people” is,

how you build up trust, which is pretty important in this setting. And if you meet people and see [that] they’re working with the individuals, with the patients, and you can see that there’s a benefit to that, then that leads to increased engagement all round.

As this example suggests, clinicians welcomed a deeper appreciation of peer engagement. Nurses and medical staff reported that learning more about the skills, practices and personal communication styles of individual peer navigators gave them greater confidence to promote the program to more clients. This exchange of insights also enabled clinical staff to communicate better about the program to their clients. Nurses who worked primarily with new and temporary migrants said that they tailored the program, referring clients to a peer navigator from a similar background. Women and heterosexual men were also referred to the peer navigators they had the most in common with. Challenges were, however, noted with maintaining program knowledge and relationships in general practices and among medical staff working fractional hours across different workplaces.

Clinic management viewed peer navigators as an additional avenue to seek feedback about their services and retain clients in care. As the manager at one clinic explained, “some of the peer navigators are clients as well … they can say hey guys actually can you, this is what people are experiencing, can we change this? [nurse FGD].” The nursing team at this clinic worked with peer navigators to stay in touch with clients if contact had been lost. In a focus group, nurses discussed an example of one woman who had temporarily moved overseas.

Her health was not good, and the peer navigator was able to message her and say you need to see a doctor. It’s not very many but there’s a significant [number of] people who would drop out of care if they didn’t have that extra support from a peer, rather than a health professional.

Upon returning to Australia the client reengaged with care.

Feedback from peer workers indicated the peer navigation program significantly improved continuity between clinical care and support groups, workshops and other peer initiatives for women and new and temporary migrants. These workers described peer navigators providing tailored and responsive support from the point of clinical care as “the missing link” in the Victorian HIV sector.

## 4. Discussion

This paper explored the perspectives of community and clinician stakeholders to better understand how a peer navigation program operating in Victoria contributed to a socially supportive, health enabling environment for people living with HIV. Participants spoke of the isolation, fears, and threats to self-worth that an HIV diagnosis can precipitate, particularly for people without connection to community. Our consideration of the enabling environment identifies how the opportunity for personal and service change peer navigation offers may facilitate stronger connection to social, community, and service contexts that can enable sustained improvements in health and quality of life.

Navigators were seen as valuable sources of reassurance and acceptance who could, together with clients, create insights to overcome obstacles HIV posed for their relationships and aspirations. Most of all, participants across all groups valued the ability of navigators to connect people living with HIV to various supports in their environment. These included healthcare and other services but primarily peer-based programs and other people living with HIV. In this sense, peer navigators were seen as revealing a socially supportive and health enabling environment for isolated or newly diagnosed people living with HIV. Although researchers have often identified how these activities encourage behaviour change relating to specific continuum of care outcomes, little research has considered their significance in improving factors related to quality of life for people living with HIV such as self-esteem, social connectedness, health, and social function or finding a sense of control and purpose in life (14, 21).

Beyond positioning peer navigation programs as a promising intervention to achieve the strategic aim of improving quality of life for people living with HIV (9), these community and clinician perspectives offer several insights for planning, implementing, and improving similar programs as well as peer responses to HIV more generally.

Our findings cast the peer navigation program as a response from a care and support sector adapting to the needs of an increasingly diverse and multifaceted community of people living with HIV. Although participants hoped peer navigators could link people from migrant backgrounds and low prevalence groups to each other and supports in the community, much of this service and community environment has been shaped by the needs and involvement of Australian-born gay men (22). Further research may reveal how intersecting forms of stigma and discrimination, gender, sexuality and cultural beliefs and backgrounds influence engagement with peer and community settings for people living with HIV from more diverse backgrounds (6, 23). This growing evidence base emphasises the importance of continued investment in community development and support structures for people living with HIV that reflect their diverse needs and experiences. Where there are no appropriate community and support structures to link service users to, the impact of peer navigation programs on quality of life may be limited.

Peer navigation programs are often thought to help people living with HIV traverse complexity in healthcare systems (14). Our findings suggest that peer navigators are skilled at identifying services and networks that are safe and non-stigmatising for people living with HIV. Often, these were peer programs and networks, however, peer navigators were able to identify a range of other services from which people living with HIV could seek help with issues ranging from migrating to Australia to managing substance misuse. Further, our findings indicate that peer navigators can help people living with HIV overcome the fear and stigma held within themselves that can prevent them from seeking support. Clinicians and peer workers both described the hesitancy, fear, and embarrassment that some people living with HIV can experience when identifying as HIV positive in care and support settings. Recent evidence reviews show that little research investigates stigma reduction interventions that target self-stigma or involve people living with HIV in their design and delivery (24, 25). As the HIV response moves to address the impact of stigma on the ability of people living with HIV to seek support our study presents peer navigation as a promising intervention to combat these manifestations and effects.

Our study draws attention to how peers coproduce and refine insights. As often identified in the peer support and self-help literature, the experiential knowledge peers use when providing support tends to lead to pragmatic solutions to the problems faced in their shared circumstances (26). In line with the observations of Salzer and Shear (27), participants identified that valuing the insights and experiences of program users in this process promotes choice, self-determination and empowerment for people who may experience marginalisation or are otherwise disempowered through hierarchical support structures. Just as Brown et al. (18) found, peer navigators acknowledged the limits of knowledge gained through their own experiences. Peer workers and navigators credited their ability to engage with many peers and the refined knowledge housed within a peer-based organisation with improving the quality of the insights they were able to produce with new program users. These findings underlie one of the advantages of peer navigation and similar programs operated by peer-based organisations as well as the importance of links back to networks and communities of experienced peers to enhance the work of peer navigators operating within non-peer or mainstream organisations.

A key strength of Living Positive Victoria’s peer navigation program was that it worked as a partnership between clinical services and the peer response in Victoria. HIV clinicians and diagnosing doctors in Australia are often unsure of how or when to refer to a peer provider (28). Our study suggests that peer navigation programs employing peers from diverse backgrounds are an appropriate service to offer people living with HIV experiencing challenges at any point in their journey.

Partnership required clinical stakeholders to place trust in Living Positive Victoria. Unlike many other healthcare settings, the clinicians in our study viewed peer organisations as credible, and appreciated the value, principals, and expertise of peer-based practices and perspectives (29–31). The partnership streamlined referral processes and facilitated exchanges of insights and awareness which could be used to enhance the client experience. As reported by peer workers this alignment strengthened continuity of care from clinical services for people living with HIV from low prevalence and culturally diverse groups. To protect or reproduce the benefits of clinical referral relationships, we recommend pursuing formal partnerships outlining and promoting program scope, organisational responsibilities, Greater and Meaningful Involvement of People living with HIV and AIDS (GIPA and MIPA) principals as well as regular opportunities for peer and clinical staff to learn about each other and share insights.

The findings of this article will be most relevant in high income, low prevalence contexts with HIV epidemics concentrated in key populations. The tradition of collaboration between affected communities, government and medical professionals in the Australian HIV response noted in our study is likely to have influenced the local care and service environment. One limitation of this study is that the perspectives of service users were not incorporated into our findings. Those closely familiar with the peer navigation program and the care and support needs of people living with HIV, including peer workers, were, however, involved in all stages of our research. Engaging program staff, planners, management and clinical stakeholders informed which communities of people living with HIV to target for involvement. Outputs from our broader program of work will refine findings and convey the experiences of women, heterosexual men and culturally and linguistically diverse new and temporary migrant gay and bisexual men who engaged with the program.

## 5. Conclusion

Our consideration of the nature of peer engagement underscores the potential for peer navigators to offer people living with HIV insights, reassurance, acceptance and belonging to overcome stigma and other barriers to community and support. These resources contribute to a socially supportive environment that can enable better health-related quality of life. The collaboration of navigators with service users and the wider peer response was thought to improve the quality of peer insights. There was also evidence to show that developing trusting referral relationships with clinicians and other service providers improved continuity of care for people living with HIV from culturally diverse and low prevalence groups. These findings allude to the wider impact of the service alignment and community engagement that peer programs can produce. We consider how these benefits can be reproduced or preserved, however, we note the need for further research and investment in community development and support initiatives that form part of this enabling environment. Where there are no appropriate community and support structures to link service users to, the impact of peer navigation programs on quality of life may be limited.

## Data availability statement

The datasets presented in this article are not readily available in order to protect the privacy of participants. Requests to access the datasets should be directed to the corresponding author.

## Ethics statement

The studies involving human participants were reviewed and approved by La Trobe University Human Research Ethics Review Board. The participants provided their written informed consent to participate in this study.

## Author contributions

TK conceived of the article concept and developed the study design under supervision of GB, AB, and SG. TK recruited participants and conducted data collection with support from SG and JH. TK conducted analysis and wrote the manuscript with supervision and feedback from AB, GB, SG, and JH. All authors contributed to the article and approved the submitted version.

## Funding

This research was supported by an investigator led research grant from ViiV Healthcare and a PhD scholarship from La Trobe University.

## Conflict of interest

This research was conducted by TK in the course of studying for a Doctor of Philosophy degree at La Trobe University.

The remaining authors declare that the research was conducted in the absence of any commercial or financial relationships that could be construed as a potential conflict of interest.

## Publisher’s note

All claims expressed in this article are solely those of the authors and do not necessarily represent those of their affiliated organizations, or those of the publisher, the editors and the reviewers. Any product that may be evaluated in this article, or claim that may be made by its manufacturer, is not guaranteed or endorsed by the publisher.
